# Heavy metal pollution of street dust in the largest city of Mexico, sources and health risk assessment

**DOI:** 10.1007/s10661-021-08993-4

**Published:** 2021-03-16

**Authors:** Anahi Aguilera, Francisco Bautista, Margarita Gutiérrez-Ruiz, Agueda E. Ceniceros-Gómez, Rubén Cejudo, Avto Goguitchaichvili

**Affiliations:** 1 Posgrado en Ciencias Biológicas, Universidad Nacional Autónoma de México, Morelia, Michoacán México; 2 Laboratorio Universitario de Geofísica Ambiental, Centro de Investigaciones en Geografía Ambiental, Universidad Nacional Autónoma de México, Morelia, Michoacán México; 3 Laboratorio de Biogeoquímica Ambiental, Facultad de Química, Universidad Nacional Autónoma de México, Mexico City, Mexico; 4 Laboratorio Universitario de Geofísica Ambiental, Instituto de Geofísica Unidad Michoacán, Universidad Nacional Autónoma de México, Morelia, Michoacán México

**Keywords:** Contamination factor, Pollution load index, Heavy metal loads, USEPA health risk assessment

## Abstract

In large industrialized cities, tons of particles containing heavy metals are released into the environment and accumulate on street surfaces. Such particles cause a potential risk to human health due to their composition and size. The heavy metal contamination levels, main emission sources, and human health risks were identified in 482 samples of street dust. Heavy metal concentrations were obtained by microwave-assisted acid digestion and ICP-OES. The results indicated that street dust in Mexico City is contaminated mainly with Pb, Zn, and Cu, according to the contamination factor and the geoaccumulation index. The pollution load index of the street dust was made with the concentrations of Pb, Zn, Cu, Cr, and Ni. The main sources of Pb, Zn, Cu, and Cr are anthropic, probably due to vehicular traffic. The highest levels of Cr and Pb in urban dust represent a health risk for children. Contamination limits were proposed for heavy metals in street dust of Mexico City. These limits might be useful to generate and apply public policies to decrease anthropic emissions of the heavy metals studied, particularly Cr and Pb.

## Introduction

In several cities around the world, air (Son et al., 2018), soils (Ihl et al., 2015), dusts (Ali et al., 2017; Men et al., 2018; Aguilera et al., 2019), and plant contamination (Aguilar-Reyes et al., 2012) by heavy metals are a serious problem that affects population health (WHO, 2014; Budai & Clement, 2018). In the case of air and soil contamination, several countries have implemented official standards since decades ago that define the maximum permissible concentrations of heavy metals; however, there is no regulation for street dust and plants.

Street dust is composed of a mixture of naturally occurring pollutants (weathering of rocks, soil erosion, leaf litter, among others) (Cortés et al., 2015) and anthropic (brake and tire wear, engine components, and exhaust emissions) (Budai & Clement, 2018; Gunawardena et al., 2014; Świetlik et al., 2015), as well as polluting particles released by industries (Aguilera et al., 2019), homes, and the weathering of urban infrastructure (Lee et al., 2018). Street dust is a sink of particulate heavy metals that are deposited on the surface of streets, sidewalks, and windows (Rahman et al., 2019). At the same time, street dust can become a source of pollutants by resuspension and washed off by stormwater runoff (Wijesiri et al., 2018).

In particular, the study of street dust is interesting because it is in closer contact with the population than particulate matter (measured at 4 m above the ground) or soils. Heavy metals in street dust can enter the human body through three routes of exposure: inhalation, ingestion, or dermal. Depending on factors such as toxicity of the element, bioavailability, concentration, etc., as well as socioeconomic issues and the person’s health status, heavy metals can generate adverse health effects (Calderón et al., 2001; Carrizales et al., 2006; Salustri et al., 2010). Therefore, establishing the levels and limits of heavy metal contamination in street dust, as well as identifying the sources of heavy metals and the risk to human health in each city, is of utmost importance to propose solution action plans.

Mexico City is one of the largest and most populous cities in the world, with high levels of contamination that cause different health problems, e.g., DNA damage, as well as respiratory and cardiovascular diseases (Son et al., 2018). The study of heavy metals in street dust, with a large number of samples, will make it possible to propose an official standard for Mexico City to define categories of action according to different contamination limits. The objectives of this study were (a) the identification of the levels of heavy metal contamination in street dust, (b) elucidate the possible sources of heavy metals, and (c) human health risk assessment by the heavy metals in street dust of Mexico City.

## Materials and methods

### Study site and sampling design

Mexico City is located in a basin at an altitude of 2240 m above sea level, with an area of 1485 km^2^. The population stands at around 8.9 million inhabitants with a population density of 5966 inhabitants/km^2^. Considering the metropolitan area, the population reaches 23,500,000 inhabitants. Moreover, 4,000,000 vehicles circulate every day, and 40,000 small and medium industries are in operation (Molina et al., 2010).

In Mexico City, there are two climatic seasons during the year: the dry winter season from November to April and the rainy season from May to October. During winter, thermal inversions are frequent, until the sun warms the cold air, around 9 or 10 am, the pollutants disperse. The prevailing winds have northeast to southwest direction; however, the “Sierra del Ajusco” prevents the passage of the wind and thus the dispersion of pollutants (Vallejo et al., 2003). An industrial center with a high population density is located in the northern part of the city. The central part includes the historical and socioeconomic center of the city, with a high urban and commercial activity. The southern area has been dominated by residential and commercial activities (Rodríguez-Salazar et al., 2011).

**Fig. 1:**
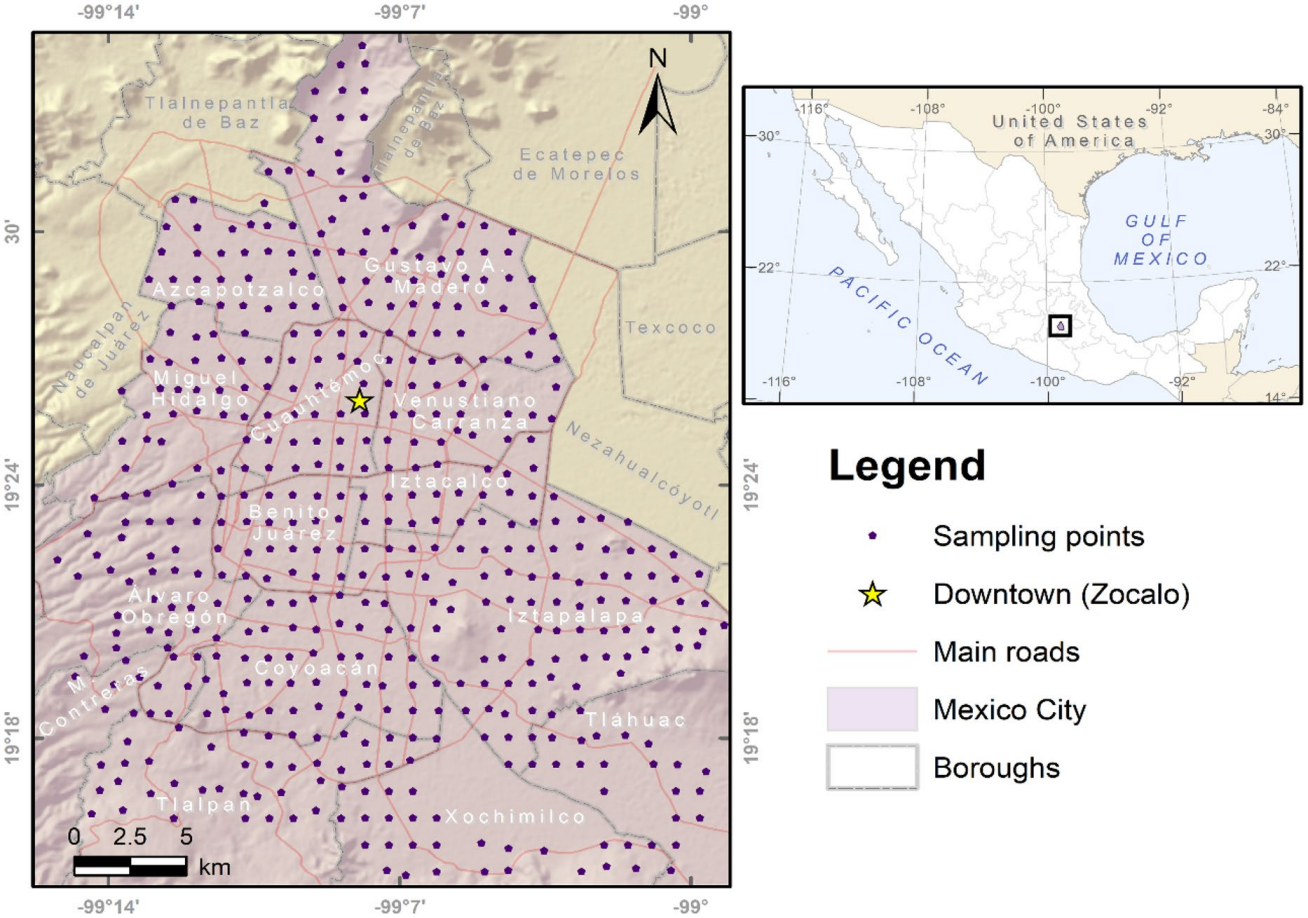
Location map of the study site and sampling points

During April and May 2017, a systematic, homogeneously distributed sampling of 482 street dust samples was carried out (Fig. 1). To collect the sample, the dust present in 1 m^2^ of the street was swept and the geographic coordinates of each site were taken. The samples were transferred to the laboratory where they were allowed to dry at room temperature and in the shade. After that, samples were sieved at 250 μm, since at this size, the particles adhere easily to the hands and can be ingested (Jadoon et al., 2018).

### Geochemical analysis and contamination levels

To determine heavy metal concentrations, 0.4 g of each sample was digested with 20 mL of concentrated HNO_3_, in an ETHOS Easy microwave digestion system (Millestone Inc) using Teflon PFA beakers. The temperature was brought to 175 ± 5 °C in approximately 5 min and was kept at that temperature for 4.5 min. After cooling, the digested samples were filtered with Whatman No. 42 paper, then transferred into 50-mL flasks, and graduated with water type A (US-EPA method 3051A). Quality controls for the acid digestion method included reagent blanks and sample duplicates. The quality assurance and quality control (QA/QC) results showed no signs of contamination or loss in any of the analyses.

Digestions and quality controls were analyzed in triplicate with an Agilent Technologies 5100 Inductively Coupled Plasma Optical Spectrometer (ICP-OES) (US: EPA method 6010C). To prepare the calibration curve, multi-elemental QCS-26R reference-certified material was used (high purity brand). Radiofrequency power (RF power) was 1.2 kW, nebulization flow 0.7 L/min, and argon plasma flow was 12.0 L/min. The detection (DL) and quantification (QL) limits are presented in Table 1. DL and QL were estimated during validation by measuring low concentration repeats, DL was calculated as 3 times the standard deviation of the repeats, and QL was calculated as 10 times the standard deviation of the same repeats.

**Table 1:** Detection (DL) and quantification (QL) limits for the elements analyzed in the wavelength used by Agilent Technologies 5100 ICP-OES

Metal	λ (nm)	DL (mg/kg)	QL (mg/kg)
Al	308.2	73.75	246.25
Ba	233.5	2.5	7.5
Ca	317.9	75	250
Co	228.6	1.25	5
Cr	267.7	1.25	5
Cu	324.7	1.25	2.5
Fe	238.2	27.5	93.75
Mg	279.0	52.5	177.5
Mn	257.6	5	15
Ni	231.6	1.25	6.25
Pb	220.3	3.75	15
V	292.4	2.5	6.25
Zn	213.8	2.5	12.5

Two contamination indexes were calculated for each heavy metal: geoaccumulation index (*I*_geo_) and contamination factor (CF), as well as the pollution load index (PLI) which is the geometric average of the five highest CF (Eq. 1–3) (Tomlinson et al., 1980).

1 $${I}_{\mathrm{geo}}={Log}_{2}({C}_{\mathrm{n}}/{1.5B}_{\mathrm{n}})$$

2 $$CF={C}_{\mathrm{n}}/{B}_{\mathrm{n}}$$

3 $$PLI=\sqrt[n]{CF1*CF2*\dots *CFn}$$

${C}_{\mathrm{n}}$ represents the concentration of the heavy metal $n$, and ${B}_{\mathrm{n}}$ is the background geochemical value. Constant 1.5 is used to compensate for natural fluctuations of the heavy metals studied and to compensate for small anthropic influences.

Generally, the background values found in healthy, non-degraded, or managed soils are used as background values; when this information is not available, the global background values for soils have been used (Kabata-Pendias, 2011) or even the minimum value found in the data under study (Declercq et al., 2019). In this research, the first decile was used because no background values have been established for street dust. We use the first decile instead of the minimum value to allow some variation and tolerance. Besides, the global background values for soils were also used, to make comparisons between the two proposals. The interpretation of the *I*_geo_ (Müller, 1979) and CF (Ihl et al., 2015) is presented in Table 2. A *PLI* value close to one indicates that the heavy metal load is close to the bottom level, while a *PLI* > 1 indicates contamination (Mehr et al., 2017; Tomlinson et al., 1980).

**Table 2:** Geoaccumulation index (*I*_geo_) and contamination factor (CF) interpretation

*I*_geo_	Interpretation
< 0	Uncontaminated
0–1	Uncontaminated to moderately contaminated
1–2	Moderately contaminated
2–3	Moderately to highly contaminated
3–4	Highly contaminated
4–5	Highly to very highly contaminated
> 5	Very highly contaminated
**CF**	**Interpretation**
< 1	Insignificant contamination
1–3	Moderate contamination
3–6	Considerable contamination
> 6	High contamination

### Sources of heavy metals

Principal component analysis (PCA) was used as a qualitative pattern recognition method, which can indicate the sources that enrich the concentrations of the heavy metals studied. The PCA reduces the data and extracts a small number of factors (principal components PC), to determine the relationships between the observed variables. The eigenvector with the longest eigenvalue is the direction of greatest variation, the second largest eigenvalue is the orthogonal direction with the next largest variation, and so on. Each PC contains information on all the elements present in a single group, while loads of each element indicate their relative contribution to the formation of the group (Rout et al., 2014).

### Human health risk by heavy metal contamination in street dust

To estimate the risk of heavy metals, present in street dust on the health of the population, the methodology developed by the United States Environmental Protection Agency (USEPA) was used. First, the estimated daily intakes for the three main exposure routes were calculated: ingestion (${\mathrm{EDI}}_{\mathrm{ing}}$), inhalation (${\mathrm{EDI}}_{\mathrm{inh}}$), and dermal contact (${\mathrm{EDI}}_{\mathrm{dermal}}$) (Eqs. 4–6), as well as the lifetime average daily dose (LADD) to estimate the carcinogenic risk (Eq. 7).

4 $${\mathrm{EDI}}_{\mathrm{ing}}=\frac{C\times IngR\times EF\times ED\times CF}{BW\times AT}$$

5 $${\mathrm{EDI}}_{\mathrm{inh}}=\frac{C\times InhR\times EF\times ED}{PEF\times BW\times AT}$$

6 $${\mathrm{EDI}}_{\mathrm{dermal}}=\frac{C\times SA\times AF\times ABS\times EF\times ED\times CF}{BW\times AT}$$

7 $$\mathrm{LADD}=\frac{C}{\mathrm{PEF}\times {\mathrm{AT}}_{\mathrm{can}}}\times \biggl(\frac{{\mathrm{CR}}_{\mathrm{child}}\times {\mathrm{EF}}_{\mathrm{child}}\times {\mathrm{ED}}_{\mathrm{child}}}{{\mathrm{BW} }_{\mathrm{child}}}+\frac{{\mathrm{CR}}_{\mathrm{adult}}\times {\mathrm{EF}}_{\mathrm{adult}}\times {\mathrm{ED}}_{\mathrm{adult}}}{{\mathrm{BW}}_{ \mathrm{adult}}}\biggr)$$

All the exposure factors used in this study are those established for reference populations (Table 3). The use of local factors could improve the reliability of the model; however, exposure factors have not been estimated yet for any Mexican City. CR is the contact (or absorption) rate. CR = IngR for ingestion, CR = InhR for inhalation, and CR = SA × AF × ABS for dermal contact. The type of CR used for each carcinogenic metal depends on the exposure route by which it can cause cancer (Table 4).

**Table 3:** Exposure factors of reference populations for human health risk assessment

Factor	Definition and units	Value		Reference
		Child	Adult	
IngR	Ingestion rate (mg/day)	200	100	USEPA (2001)
InhR	Inhalation rate (m^3^/day)	7.63	12.8	Li et al. (2001)
PEF	Particle emission factor	1.36E + 09	1.36E + 09	USEPA (2001)
SA	Surface of exposed skin area (cm^2^)	2800	5700	USEPA (2001)
ABS	Dermal absorption factor	0.001	0.001	USEPA (2001), Ali et al. (2017)
AF	Skin adherence factor (mg/cm^2^)	0.2	0.07	USEPA (2001)
ED	Duration of exposure (years)	6	24	USEPA (2001)
EF	Frequency of exposure (days/year^1^)	350	350	Zheng et al. (2010)
AT	Average time non-carcinogens (days)	ED*365	ED*365	USEPA (1989)
Atcan	Average time for carcinogens (days)	70*365	70*365	USEPA (1989)
BW	Body weight (kg)	15	70	Zheng et al. (2010), Mohmand (2015), Kurt-Karakus (2012)
C	Heavy metal concentration (mg/kg)	This study		
CF	Conversion factor (kg/mg)	1 × 10^−6^		Li et al. (2001)

**Table 4:** Reference doses (RfD) and cancer slope factor (CSF) for each route of exposure

	Oral RfD	Inhalation RfD	Dermal RfD	Oral CSF	Dermal CSF	Inhalation CSF
Co	2.00E-02	5.71E-06	1.60E-02			9.80E + 00
Cr	3.00E-03	2.86E-05	6.00E-05			4.20E + 01
Cu	4.00E-02	4.02E-02	1.20E-02			
Fe	8.40E + 00	2.20E-04	7.00E-02			
Mn	4.60E-02	1.43E-05	1.85E-03			
Ni	2.00E-02	2.06E-02	5.40E-03			8.40E-01
Pb	3.50E-03	3.52E-03	5.25E-04	0.0085		4.20E-02
V	7.00E-03	7.00E-03	7.00E-05			
Zn	3.00E-01	3.00E-01	6.00E-02			

Risk ratios for ingestion, inhalation, and dermal contact (${\mathrm{HQ}}_{\mathrm{ing}/\mathrm{inh}/\mathrm{derm}}$) were obtained by dividing the $EDI$ by the reference dose ($RfD$) as shown in Eq. 8:

8 $${\mathrm{HQ}}_{\mathrm{ing}/\mathrm{inh}/\mathrm{derm}}=\frac{{\mathrm{EDI}}_{\mathrm{ing}/\mathrm{inh}/\mathrm{derm}}}{\mathrm{RfD}}$$

The hazard index (HI) represents the sum of the $HQ$s for the three routes of exposure. If HI is greater than 1, non-carcinogenic effects on the health of the population could occur; if it is less than 1, the opposite would be expected (USEPA, 2001).

For carcinogenic elements, the incremental lifetime cancer risk (ILCR) is commonly calculated using the following equation:

9 $$\mathrm{ILCR}=\mathrm{LADD}\times \mathrm{CSF}$$

The accepted or tolerable risk is in the range of 1E-06 to 1E-04 (USEPA, 2001). These values indicate that an additional case in a population of 1,000,000 and 10,000 people is acceptable (Lu et al., 2014).

## Results

### Contamination levels by heavy metals in street dust in Mexico City

All the analyzed elements (Ba, Co, Cr, Cu, Mn, Ni, Pb, Ti, V, Zn, Al, Ca, Fe, and Mg) had an asymmetric distribution (not normal) according to the Shapiro test; therefore, the median is used as a reference of central tendency. The median concentrations of the elements decreased in the order Ca > Al > Mg > Fe > Ti > Zn > Mn > Ba > Pb > Cu > Cr > Ni > V > Co (Table 5). The first five elements and the Mn are considered as major elements because they are more abundant in the Earth’s crust, while the rest are called trace elements due to their small concentrations.

**Table 5:** Statistical summary of the heavy metal concentrations in mg/kg and background values

Heavy metal	*n*	Min	Max	Median	Mean	CV	Background decile 1	Global soils background value^a^
Ba	482	41.2	446.0	122.5	128.2	0.4	77.4	460.0
Co	482	2.5	82.4	7.5	7.4	0.6	5.0	11.3
Cr	482	15.0	441.0	43.7	51.4	0.7	28.8	59.5
Cu	482	6.2	847.1	81.2	99.7	0.8	36.2	38.9
Mn	482	100.0	990.5	223.7	235.2	0.3	166.3	488.0
Ni	482	13.7	148.7	35.0	36.3	0.4	22.5	29.0
Pb	481	8.8	1907.8	101.2	128.2	1.0	38.7	27.00
Ti	422	96.3	1677.1	365.5	412.3	0.5	254.9	7038.0
V	482	11.2	160.0	26.2	26.8	0.3	18.8	129.0
Zn	482	18.7	4827.6	229.9	280.7	1.0	113.8	70.0
Al	482	2823.6	59,285.2	9045.5	10,853.3	0.6		
Ca	482	1450.0	261,937.5	51,411.8	57,430.1	0.6		
Fe	482	653.3	61,326.8	3817.0	5722.2	1.0		
Mg	482	984.8	30,780.7	6484.5	6948.9	0.5		

*Min* minimum, *Max* maximum, *CV* coefficient of variation

^a^Kabata-Pendias (2011)

Both the lack of normality in the frequency distribution and the wide differences between the mean and the median are considered qualitative indicators of the anthropic origin of the elements. The elements that showed the greatest differences between the mean and the median were Fe, Pb, Cu, Zn, Al, Cr, V, and Ca. Besides, the highest coefficients of variation corresponded to Fe, Pb, Zn, Cu, and Cr. These characteristics are the first indications that the concentrations of these elements have been enriched by the anthropic activities in Mexico City. This is logical due to the widespread use of these elements. Especially, these elements are related to the vehicle fleet, body materials, auto part wear, brake lining, motor body, wear and tear of the tire, and other parts (Rahman et al., 2019; Jiang et al., 2018). 

Table 6 also summarizes the descriptive statistics of the loadings of the analyzed elements. The loadings were obtained by multiplying the concentrations of each element by the amount of dust, in kg, present on 1 m^2^ of the surface. None of the loads had a normal distribution, according to the Shapiro test. The medians decreased in the same order as the concentrations of the elements: Ca > Al > Mg > Fe > Ti > Zn > Mn > Ba > Pb > Cu > Cr > Ni > V > Co.

**Table 6:** Statistical summary of the heavy metal loads in mg/m^2^

Heavy metal load	*n*	Min	Max	Median	Mean	CV
Ba	482	0.47	18.90	5.47	5.74	0.55
Co	482	0.02	3.42	0.31	0.34	0.73
Cr	482	0.18	15.10	1.91	2.28	0.74
Cu	482	0.12	33.12	3.50	4.45	0.87
Mn	482	0.92	33.56	9.82	10.69	0.53
Ni	482	0.14	9.67	1.45	1.70	0.68
Pb	481	0.12	52.78	4.19	5.46	0.93
Ti	422	0.96	109.31	15.49	18.69	0.71
V	482	0.11	7.82	1.12	1.22	0.56
Zn	482	0.42	195.29	9.99	12.25	1.00
Al	482	33.16	3346.18	399.23	481.67	0.73
Ca	482	53.39	17,132.74	2138.60	2615.96	0.82
Fe	482	6.88	2213.50	160.70	260.51	1.13
Mg	482	13.89	1950.83	283.55	335.01	0.74
Dust load (g/m^2^)	482	5.4	173.3	43.00	46.40	0.5

*Min* minimum, *Max* maximum, *CV* coefficient of variation

Table 5 shows the global background values for soils, which were used in this study, along with the first decile of the frequency distribution of each element. Comparing both values highlights the fact that the world background values of soils for Ba, Mn, Ti, and V are more than three times greater than the values proposed in this article (first decile); in the case of Ti, it is 27 times greater. This is because these elements are abundant in soils; however, they are not so abundant in street dust. The opposite occurs for Pb and Zn, the first decile of the frequency distribution is greater than the global background value for soils, which indicates that the Pb and Zn of street dust come mainly from anthropic sources.

### Contamination factor

**Fig. 2:**
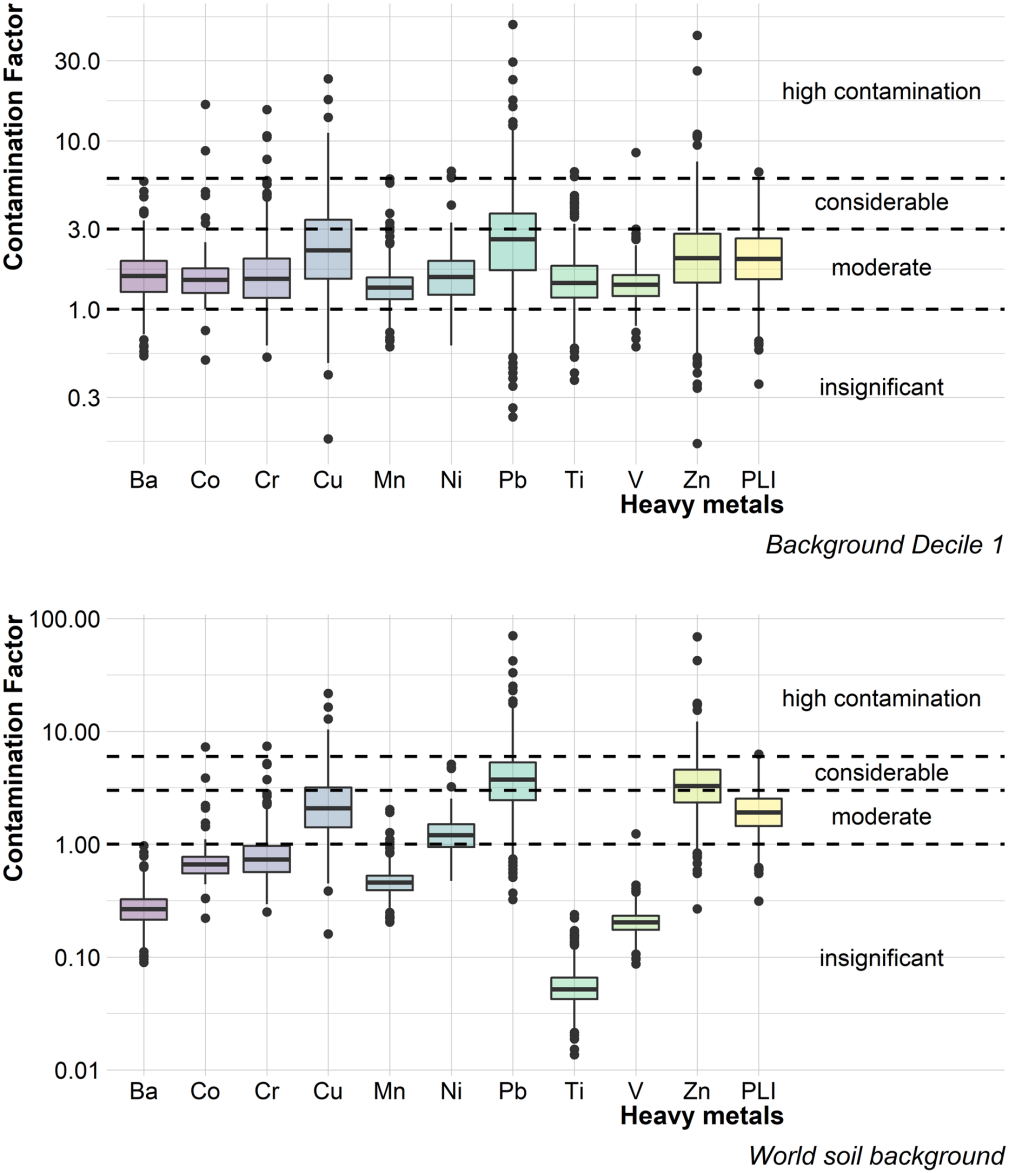
Boxplots for heavy metal contamination factors using the first decile (top image) and the global background value for soils (Kabata-Pendias, 2011) (bottom image) as the background value

When the first decile was considered as the background value of street dust from Mexico City, most of the data for all the elements were located at the threshold of moderate contamination (Fig. 2). The Pb, Cu, and Zn concentrations had the highest CF, the extreme CF values of these elements (Pb, Cu, and Zn) were located in the category of high contamination.

When considering as a background value the one established worldwide for soils, then a greater variation in the levels of contamination was observed, between elements. The elements with the highest CFs were Pb and Zn, with more than 25% of the data in the category of considerable contamination, followed by Cu and Ni with ~50% of the data in the moderate contamination category. More than 75% of the Cr, Co, and Mn data were located in the insignificant contamination category, and practically, all the Ba, Ti, and V data were found in the same category (insignificant contamination).

In the case of the PLI for the five elements with the highest CF (Pb, Cu, Zn Cr, and Ni), more than 50% of the data had a value greater than 1, both for the first decile and for the global background value for soils, this indicates that street dust from Mexico City is contaminated by heavy metals, regardless of the background value used in this study.

It is difficult to establish the (natural) background values of the elements when it comes to such a mobile matrix, i.e., street dust, located in urban sites full of anthropic activities. Background values of soils may be inappropriate because they are not the only source of heavy metals in street dust and because the particle size at which both matrices are analyzed is very different. On the other hand, the results of this study show that the use of the first decile can be limiting by homogenizing the variation for the contamination factors.

### Geoaccumulation index

One of the benefits of the geoaccumulation index, regarding the contamination factor, is that it admits small variations in the background values. When using the first decile as the background value, it was observed that about 50% of the Pb, Zn, and Cu data were found in the category of *uncontaminated to moderately contaminated*; for the rest of the elements, with exception of Mn and V, just over 25% of the data was located in that category. Only Mn and V had most of the data in the *uncontaminated* category.

On the other hand, when considering the global background values for soils, the level of Pb and Zn contamination increased, compared with what was found with the first decile, with more than 25% of the data in the category of *moderate contamination*. Next, about 50% of the Cu data was in the *uncontaminated to moderately contaminated* category. All other elements were located as *uncontaminated*, in ascending order Ti < V < Ba < Mn < Co < Cr < Ni.

The most polluting elements in urban Mexico City dust were Pb and Zn, followed by Cu, regardless of the background value used in this work. However, when using the global background value for soils, the level of contamination was higher. Furthermore, even when the median was at an *uncontaminated to moderately contaminated* level when the first decile was used, and *moderately contaminated* when the global background value for soils was used; in both cases, some sampled sites reached the category of highly to very highly contaminated by Pb and Zn.

Heavy metals with the highest contamination in street dust from Mexico City were Pb, Zn, and Cu, secondly Ni and Cr; considering CF and Igeo, with two different background levels (global value for soils and the first decile). Recognizing that these elements are related to vehicular traffic (Budai & Clement, 2018; Świetlik et al., 2015; Gunawardena et al., 2014), this sector could be pointed out as one of the most, if not the most influential, in heavy metal contamination in street dust in Mexico City. The PLI (Fig. 2) indicated that the level of contamination by the most polluting heavy metals (Pb, Zn, Cu, Ni, and Cr) is moderate, regardless of the background value considered (first decile and world soil background).

### Proposal of contamination limits

The need to establish levels of regulation of heavy metal concentrations in street dust is evident; however, it is a complicated issue, starting with the difficulties of establishing a reference value or background value that represents a state of non-contamination. In the present work, we have compared the CF and Igeo using two background levels: (1) the first decile of the frequency distribution and (2) the global background value for soils. Each has its advantages and disadvantages; however, we consider that it is more appropriate to use the first decile as it is a specific value for Mexico City.

Using the first decile as the background value, contamination limits were established taking the CF as a reference (Table 7). The categories proposed for these limits are those used by Galán and Romero (2008) for Spain: (1) reference value (CF < 1): equivalent to the background value, indicates that there is no contamination below that level. (2) Recommended investigation level (CF: 1–3): there could be an insignificant to moderate degree of contamination, so it is recommended to be alert and, if possible, analyze bioavailable concentrations for the human body. (3) Mandatory investigation level (CF: 3–6): a considerable to a high level of contamination is assumed; therefore, it is considered mandatory to investigate bioavailable concentrations or to carry out a sequential chemical extraction. (4) Intervention level (CF > 6): a high level of contamination is assumed; therefore, mitigation actions must be carried out to reduce contamination.

**Table 7:** Proposed contamination limits for heavy metals in street dust in Mexico City

Heavy metal	Reference value	Recommended investigation level	Mandatory investigation level	Intervention level
	(mg/kg)			
Ba	77.4	77–232	232–465	> 465
Co	5.0	5–15	15–30	> 30
Cr	28.8	29–86	86–173	> 173
Cu	36.2	36–109	109–217	> 217
Mn	166.3	166–499	499–998	> 998
Ni	22.5	23–67	67–135	> 135
Pb	38.7	39–116	116–232	> 232
Ti	254.9	255–765	765–1529	> 1529
V	18.8	19–56	56–113	> 113
Zn	113.8	114–341	341–683	> 683

The existence of a moderate level of contamination in Mexico City highlights the need to regulate heavy metal concentrations in street dust. The contamination limits are a simple first proposal to start regulating the concentrations of heavy metals in Mexico City. Following the contamination limits proposed in this article, it would be advisable to carry out more exhaustive analyzes on anthropic metals (Pb, Zn, Cu, Ni, and Cr); it is mainly recommended to analyze bioavailable concentrations for humans. Although the contamination limits are based on total concentrations, toxicological analyses are also required. The proposed categories (Galán & Romero, 2008) allow having a reference on the action measures required at each level of contamination, which is very useful for decision-makers.

### Main sources of heavy metals in street dust of Mexico City

**Fig. 3:**
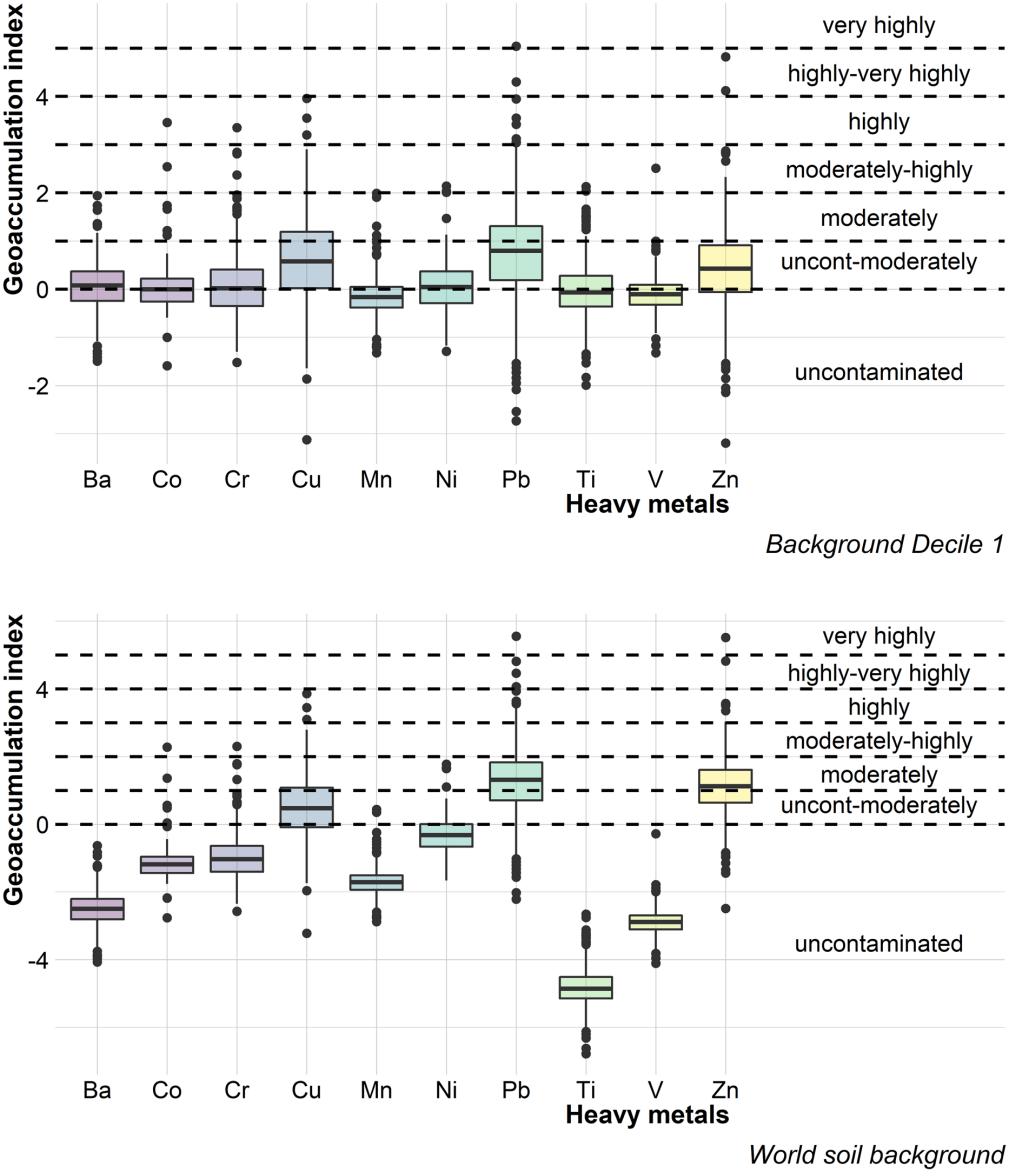
Boxplots for heavy metal geoaccumulation index using the first decile (top image) and the global background value for soils (Kabata-Pendias, 2011) (bottom image) as the background value

**Fig. 4:**
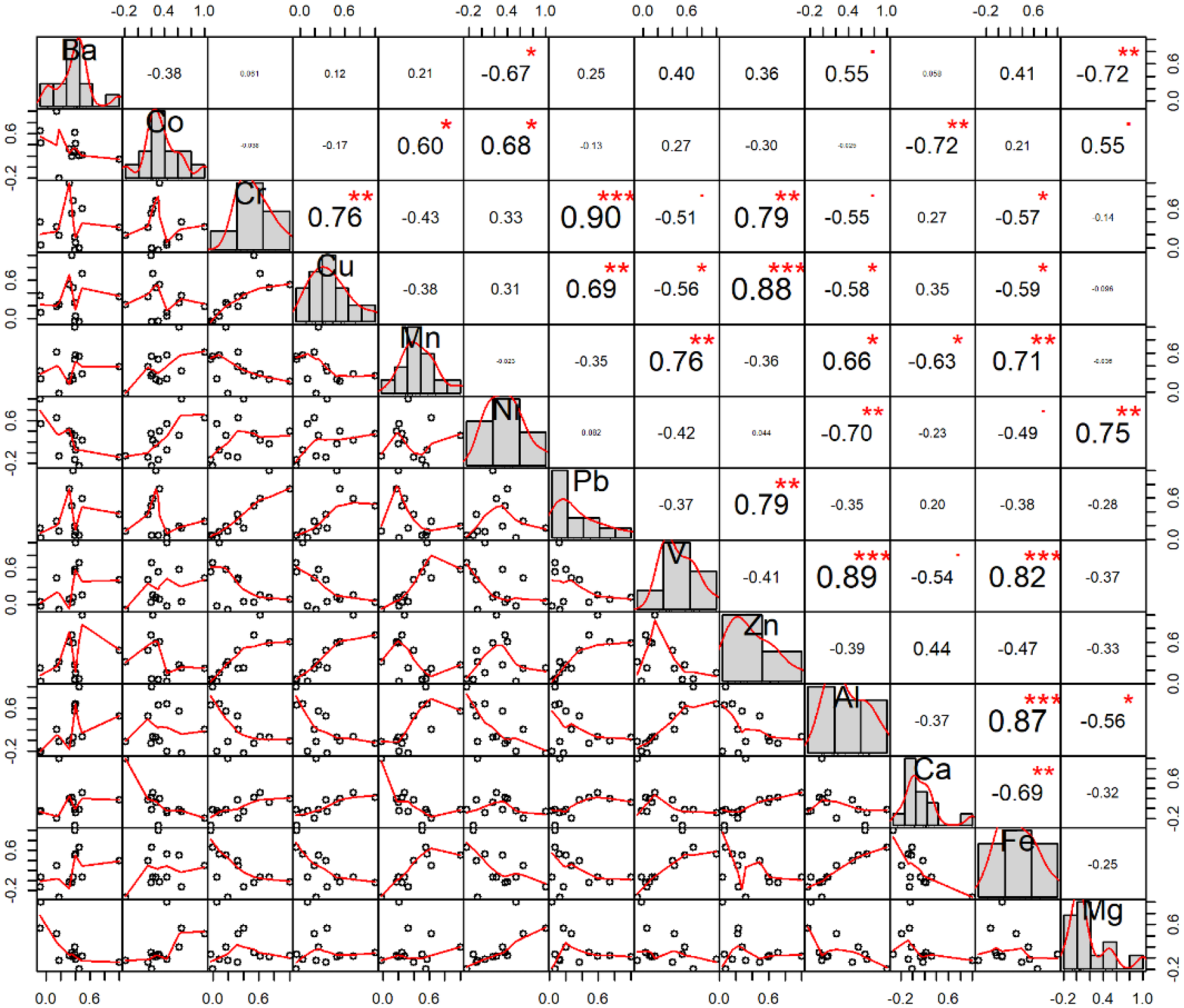
Spearman correlation coefficients at the top, * indicates statistical significance with *p* = 0.05. In the lower part, the scatter plots of the corresponding pairs of variables can be seen

Correlation coefficients have been widely used to identify those elements that came from the same sources. In this study, Spearman’s method was used as the correlation method, since none of the elements had a normal distribution. Pb-Cr-Cu-Zn concentrations showed a strong and significant correlation (Fig. 3), with a coefficient greater than 0.6. The Mn-V-Al-Fe also maintained a strong and significant correlation between them. On the other hand, Fe had a strong and significant, but indirect, relationship with Ca, Cr, Cu, and Ni. Also, other significant correlations were found between pairs of elements that can be reviewed in detail in Fig. 4.

The principal component analysis (PCA) was applied on log-transformed data to reduce the influence of high values because data were not normally distributed. Moreover, data were centered and scaled before the analysis was performed. PCA has been a useful method to identify anthropic pollution sources of heavy metals in soils (Liao et al., 2018) and dust (Chen et al., 2014).

The first two PC had eigenvalues greater than one; therefore, they were extracted and are shown in Table 8. Mg, Ca, and Ni concentrations were excluded from the analysis due to poor representability. PC1 explained 40.41% of data variance and it was dominated by Fe, Mn, V, and Al; the elements identified as the natural ones. On the other side, PC2 explained 25.9% of the data variance and it was dominated by the anthropic elements: Pb, Cr, Zn, and Cu. These four metals were the most polluting elements of the street dust in Mexico City (Cr, Cu, Pb, and Zn), according to the contamination indexes previously calculated; this also suggests that they are elements of anthropic origin.

**Table 8:** Component matrix for data of Mexico City street dust

Elements	Component 1	Component 2
	Fe, Mn, V, Al	Pb, Cr, Zn, Cu
Ba	0.31	0.16
Co	0.31	0.04
Cr	0.18	0.40
Cu	0.16	0.43
Mn	0.38	−0.08
Pb	0.20	0.44
Ti	0.30	−0.33
V	0.38	−0.17
Zn	0.20	0.43
Al	0.35	−0.29
Fe	0.42	−0.19
Eigenvalue	4.45	2.85
Cumulative variance (%)	40.41	66.29

It has been recognized in the literature that Cu, Pb, Zn, and Cr are traffic-related metals (Budai & Clement, 2018; Świetlik et al., 2015; Gunawardena et al., 2014). Although in varying quantities, Cu and Pb emissions are known to originate from brake wear, Pb can also come from the loss of lead wheel weights, while Zn is mainly emitted by tire and brakes wear, as well as from diesel exhaust emission, and some Zn compounds are used as additives for motor oil (Budai & Clement, 2018; Świetlik et al., 2015). Cr can originate from exhaust emissions (Gunawardena et al., 2014).

In the present study, the results of the Spearman correlation coefficients and PCA showed an association between Pb and Cr; this has also been previously observed in studies on street dust (Lee et al., 2018; Legalley & Krekeler, 2013). Lee et al. (2018) concluded that lead chromate in dust particles originates from yellow street paint; furthermore, they argued that the paint containing lead chromate is one of the largest sources of Pb contamination in street dust; therefore, lead chromate paint could be a probable source of Pb and Cr in Mexico City street dust. Once the lead chromate begins to deteriorate due to abrasion, humidity, and temperature, as well as the exposure of the pigments to light (it is a photo-sensitive pigment), paint and pigments crack, peel, and turn to chalk, mobilizing metal particles into the urban environment (Legalley & Krekeler, 2013).

In Mexico City, only one previous study on heavy metals in street dust has been carried out, whose sampling took place in 2011. Compared with that study, the same heavy metals continue to have the highest degree of contamination: Cr, Cu, Pb, and Zn (Delgado et al., 2019). In comparison with Mexico City soils, previous studies also identified Pb, Cu, and Zn as anthropic elements; however, Cr was considered as a natural element in soils, while in street dust, it seems to be anthropic. In the case of the present study, the very strong Spearman’s correlation coefficient (*r* = 0.9) found between Pb, which is consensually anthropic, and Cr indicates that the origin of Cr in street dust is mainly anthropic.

### Human health risk by heavy metal contamination in street dust

The evaluation of the health risk using the USEPA methodology showed that the mean concentrations of the elements were within the level considered to be safe for the population health (children and adults). This indicates that there is no risk of developing adverse health effects due to exposure to the analyzed heavy metals (HI < 1) (Table 9).

**Table 9:** Hazard indexes (HI) for children and adults and risk index (RI)

Heavy metal	Minimum	Maximum	Median	Mean	Standard deviation	Coefficient of variation
	Children					
Co	1.76E-03	5.81E-02	5.28E-03	5.26E-03	3.12E-03	5.94E-01
Cr	7.30E-02	2.15E + 00	2.13E-01	2.50E-01	1.67E-01	6.67E-01
Cu	2.02E-03	2.73E-01	2.62E-02	3.22E-02	2.45E-02	7.60E-01
Fe	2.39E-03	2.25E-01	1.40E-02	2.10E-02	2.13E-02	1.02E + 00
Mn	3.22E-02	3.19E-01	7.21E-02	7.58E-02	2.54E-02	3.34E-01
Ni	8.88E-03	9.61E-02	2.26E-02	2.35E-02	8.98E-03	3.83E-01
Pb	0.00E + 00	7.10E + 00	3.77E-01	4.75E-01	5.01E-01	1.05E + 00
V	2.63E-02	3.74E-01	6.13E-02	6.27E-02	2.06E-02	3.29E-01
Zn	8.10E-04	2.09E-01	9.94E-03	1.21E-02	1.27E-02	1.05E + 00
Sum	2.60E-01	9.39E + 00	8.32E-01	9.58E-01	6.59E-01	6.88E-01
	Adults					
Co	2.28E-04	7.54E-03	6.85E-04	6.82E-04	4.05E-04	5.94E-01
Cr	8.28E-03	2.44E-01	2.41E-02	2.84E-02	1.89E-02	6.67E-01
Cu	2.17E-04	2.94E-02	2.82E-03	3.46E-03	2.63E-03	7.60E-01
Fe	5.40E-04	5.07E-02	3.16E-03	4.73E-03	4.81E-03	1.02E + 00
Mn	4.17E-03	4.14E-02	9.34E-03	9.82E-03	3.28E-03	3.34E-01
Ni	9.55E-04	1.03E-02	2.43E-03	2.52E-03	9.67E-04	3.83E-01
Pb	0.00E + 00	7.67E-01	4.07E-02	5.13E-02	5.41E-02	1.05E + 00
V	3.08E-03	4.38E-02	7.18E-03	7.34E-03	2.41E-03	3.29E-01
Zn	8.70E-05	2.25E-02	1.07E-03	1.31E-03	1.37E-03	1.05E + 00
Sum	2.92E-02	1.03E + 00	9.57E-02	1.10E-01	7.26E-02	6.63E-01
	Risk index					
Co	2.82E-08	9.30E-07	8.45E-08	8.42E-08	5.00E-08	5.94E-01
Cr	7.25E-07	2.13E-05	2.11E-06	2.48E-06	1.66E-06	6.67E-01
Ni	1.33E-08	1.44E-07	3.38E-08	3.51E-08	1.35E-08	3.83E-01
Pb	0.00E + 00	1.11E-07	5.88E-09	7.42E-09	7.83E-09	1.06E + 00
Sum	1.00E-06	2.20E-05	2.00E-06	2.64E-06	1.70E-06	6.45E-01

The average HI of Cr and Pb for children was the closest to the safe limit, being in the order of E-01; at that level, it has been reported that ailments of different types can be triggered (Jadoon et al., 2018). Furthermore, the maximum HIs of Cr and Pb can indeed represent a health risk for children exposed to these sites, since they exceed the threshold considered safe. Therefore, attention should be paid to the concentrations of these elements present in street dust; as a conservative action, maximum values should be considered.

The maximum IR (2.13E-05) and average (2.48E-06) of Cr are within the tolerable risk, which means that ~2 cases of cancer in a population of 100,000 people may occur for the maximum RI, and 2.5 cases in a population of 1 million people may occur for the average RI. However, it depends on the oxidation state, since only Cr (IV) is carcinogenic, while in this study, the total concentrations were used for the risk assessment. In another study of Cr in street dust, it was found that around 45% of the Cr present in the sample corresponds to Cr (VI) (Lee et al., 2018); if the same occurs for Mexico City, the risk would be reduced in more than a half; however, this should be corroborated in the future.

## Conclusions

Based on a striking and representative number of samples, the contamination factor and the geoaccumulation index showed that the street dust in Mexico City is moderately contaminated by Pb, Zn, and Cu. In addition to these three elements, Cr and Ni, together, were part of the polluting load of street dust, which indicated that more than 90% of Mexico City was contaminated (polluting load index higher than one). Moreover, the maximum values of Cr and Pb concentrations could represent a health risk for children in Mexico City. These results highlight the necessity to monitor and regulate the heavy metals in the street dust; to the best of our knowledge, this regulation is not made anywhere in the world, at the moment.

Cu, Cr, Pb, and Zn in the street dust of Mexico City must have a similar origin since those elements were associated; we assumed they must be anthropic probably due to vehicular traffic, Cr and Pb could come from the lead chromate used in the yellow paint, Cu is known to originate from brake wear, Zn is emitted by tire and additives to motor oil, and Cr can originate from exhaust emissions. Fe, Mn, V, and Al must also have a common origin; those can come from natural sources or a mix of natural and anthropic sources. In future studies, it will be important to analyze the possible sources identified in this study; this will be very useful to control the emissions of heavy metals in Mexico City.

With the idea of improving the health of the population of the largest city in the country, we proposed categories of action according to the contamination levels identified by the contamination factor; we hope these contamination limits will help to launch public policies to decrease the polluting load of heavy metals in street dust of Mexico City. Government actions are needed around the reduction of emissions, a street dust managing program, and citizen cleaning campaigns, as well as education campaigns on potential health problems due to contact with street dust.

## Data Availability

Data is available to whoever requests it.
