# Correction to: Heavy metal pollution of street dust in the largest city of Mexico, sources and health risk assessment

**DOI:** 10.1007/s10661-021-09344-z

**Published:** 2021-07-31

**Authors:** Anahi Aguilera, Francisco Bautista, Margarita Gutiérrez-Ruiz, Agueda E. Ceniceros-Gómez, Rubén Cejudo, Avto Goguitchaichvili

**Affiliations:** 1 Posgrado en Ciencias Biológicas, Universidad Nacional Autónoma de México, Morelia, Michoacán México; 2 Centro de Investigaciones en Geografía Ambiental, Laboratorio Universitario de Geofísica Ambiental, Universidad NacionalAutónoma de México, Morelia, Michoacán México; 3 Laboratorio de Biogeoquímica Ambiental, Facultad de Química, Universidad Nacional Autónoma de México, Mexico City, Mexico; 4 Laboratorio Universitario de Geofísica Ambiental, Instituto de Geofísica Unidad Michoacán, Universidad Nacional Autónoma de México, Morelia, Michoacán México

## Correction to: Environ Monit Assess (2021) 193: 193 10.1007/s10661-021-08993-4

The contribution section and ORCIDs of the authors were missing in the original version of this article.

The missing contribution statement is shown below and ORCIDs for each authors were added in the author group section above.

